# Prognostic analysis of patients with stage IIIC1p cervical cancer treated by surgery

**DOI:** 10.1186/s12957-023-03076-9

**Published:** 2023-06-21

**Authors:** Xiang Fan, Yifei Wang, Ni Yang, Pengfeng Zhu

**Affiliations:** Changzhou Maternal and Child Health Care Hospital, Changzhou Medical Center, Nanjing Medical University, Changzhou, 213000 Jiangsu China

**Keywords:** Cervical cancer, Stage IIIC1p, Prognosis, Overall survival, Disease-free survival

## Abstract

**Background:**

Cervical cancer (CC) is one of the most common gynaecologic malignancies. The prognosis of stage IIIC1p cervical cancer patients treated by surgery is heterogeneous. Therefore, the aim of this study was to analyse the factors influencing the prognosis in such patients.

**Methods:**

From January 2012 to December 2017, 102 patients with cervical cancer who underwent surgical treatment in the Department of Gynaecology and Tumours, Changzhou Maternal and Child Health Hospital, and had pelvic lymph node metastasis confirmed by pathology were analysed retrospectively. All patients underwent radical hysterectomy with/without oophorectomy with pelvic lymphadenectomy with/without para-aortic lymphadenectomy. Clinical data was collected including age, surgical method, ovarian status, intraoperative blood loss, perioperative complications, tumour size, pathological type, depth of stromal invasion (DSI), whether the lymphatic vascular space was infiltrated, number of pelvic lymph node metastases, location of pelvic lymph node metastases, total number of lymph nodes resected, lymph node ratio (LNR), nature of vaginal margin, whether parametrium was involved, postoperative adjuvant therapy, preoperative neutrophil–lymphocyte ratio (NLR) and prognostic information of patients. Survival curves for overall survival (OS) and disease-free survival (DFS) were plotted using the Kaplan–Meier method, and the difference between the survival curves was tested using the log-rank test. Univariate and multivariate COX regression models were used to assess the factors associated with overall survival and disease-free survival in patients with stage IIIC1p cervical cancer. Nomogram plots were constructed to predict OS and DFS, and the predictive accuracy of the nomograms was measured by Harrell’s C-index and calibration curves.

**Results:**

A total of 102 patients with stage IIIC1p cervical cancer were included in the study, and the median follow-up time was 63 months (range from 6 to 130 months). The 5-year OS was 64.7%, and the 5-year DFS was 62.7%. Multivariate analysis showed that no postoperative adjuvant therapy, LNR > 0.3 and NLR > 3.8 were independent risk factors for OS and DFS in patients with stage IIIC1p cervical cancer.

**Conclusions:**

Patients with stage IIIC1p cervical cancer have a poor prognosis. Lower OS and DFS were associated with no postoperative adjuvant therapy, LNR > 0.3 and NLR > 3.8.

## Introduction

Cervical cancer (CC) is one of the most common gynaecologic malignancies, and its specific treatment is closely related to tumour stage. Surgical treatment is the main treatment modality for early cervical cancer, and radical hysterectomy via open or minimally invasive surgery was recommended by the National Comprehensive Cancer Network (NCCN) in 2017 and the European Society of Gynaecological Oncology/European Society for Radiotherapy and Oncology/European Society of Pathology (ESGO/ESTRO/ESP) in 2018 [1, 2]. Minimally invasive surgery is also widely used due to less bleeding, small abdominal incision scar, rapid postoperative recovery, short hospital stay and comparable efficacy to that of open surgery. A retrospective study by Pecorino et al. [3] also showed that there was no significant difference in mortality and recurrence rate between patients undergoing open surgery and minimally invasive surgery, and there was no significant difference in intraoperative and postoperative morbidity. However, the status of minimally invasive surgery has been challenged by the results of the prospective, phase III Laparoscopic Approach to Cervical Cancer (LACC) trial [4]. The results indicate that the 4.5-year disease-free survival (DFS) of patients who underwent open surgery was 96.5%, while the 4.5-year DFS of patients who underwent minimally invasive surgery was 86% [4]. After the LACC trial, Bogani et al. [5] retrospectively analysed the role of minimally invasive radical hysterectomy in cervical cancer through a literature review and clarified the potential risk factors for the adverse effects of minimally invasive surgery on the survival of patients with cervical cancer.

With the development of tumour diagnosis and treatment technology, tumour staging is also in the process of change. Lymph node positivity is one of the high-risk factors affecting the prognosis of patients with cervical cancer and can be used as a reference point for guiding adjuvant therapy for cervical cancer [6]. In 2018, the Federation International of Gynaecology and Obstetrics (FIGO) included lymph node metastasis in stage IIIC [r (imaging) and p (pathology)], and patients with pelvic lymph node metastasis or para-aortic lymph node metastasis were classified as stage IIIC1 and stage IIIC2, respectively [7]. The *NCCN Clinical Practice Guidelines for Cervical Cancer, 1st Edition, 2022* recommends that patients with pelvic lymph node metastasis indicated by imaging should be treated with concurrent chemoradiotherapy directly [8]. However, we found in practice that the new FIGO staging system has some shortcomings in clinical application. For example, the stage IIICr cervical cancer cohort includes some patients who actually do not have lymph node metastasis because not all enlarged lymph nodes suggested by imaging are true metastases. In addition, abdominal computerized tomography (CT), magnetic resonance imaging (MRI), positron emission tomography-computerized tomography (PET-CT), and other imaging methods differ in the accuracy of judging whether there is lymph node metastasis [9]. Therefore, the accuracy of imaging staging in the diagnosis of lymph node metastasis is not ideal, which may limit the study of prognosis to a certain extent.

Based on the above reasons, this study retrospectively analysed cervical cancer patients with pelvic lymph node metastasis confirmed by pathology after radical hysterectomy and comprehensively evaluated the factors affecting the prognosis of patients with stage IIIC1p cervical cancer.

## Materials and methods

### Patients

A total of 102 patients with cervical cancer who underwent extensive hysterectomy with/without oophorectomy with pelvic lymphadenectomy with/without para-aortic lymphadenectomy in Changzhou Maternal and Child Health Hospital from January 2012 to December 2017 and had pelvic lymph node metastasis confirmed by pathology were selected. According to FIGO 2018 cervical cancer staging, all diagnoses were revised to cervical cancer stage IIIC1p (preoperative staging was based on FIGO 2009: 44 cases of IB1, 25 cases of IB2, 17 cases of IIA1, 16 cases of IIA2). The clinical features of all patients were collected, including age, surgical method, ovarian status, intraoperative blood loss, perioperative complications, tumour size, pathological type, depth of stromal invasion, whether the lymphatic vascular space was infiltrated, number of pelvic lymph node metastases, location of pelvic lymph node metastases, total number of lymph nodes resected, lymph node ratio (LNR), nature of vaginal margin, whether parametrium was involved, whether postoperative adjuvant therapy was performed, neutrophil–lymphocyte ratio (NLR) and survival information of all patients. The major perioperative complications included urinary retention, ureteral stent placement due to hydronephrosis, intestinal obstruction and deep vein thrombosis. Patient eligibility criteria of this study included the following: (1) primary cervical squamous cell carcinoma, adenocarcinoma, or adenosquamous carcinoma confirmed by pathological diagnosis; (2) the preoperative diagnosis was IB-IIA according to 2009 FIGO cervical cancer staging; (3) preoperative imaging did not suggest lymph node metastasis; and (4) results were reviewed by two pathologists. Patients who met the following criteria were excluded from this study: (1) complicated with other malignant tumours and serious internal and surgical diseases; (2) lost to follow-up; (3) source of tumour metastasis in other sites; and (4) lymphatic metastasis in addition to pelvic lymph nodes. All patients had been fully informed of the relevant surgical risks before surgery and signed the informed consent form for surgery. All 102 patients were operated on by the same surgeon and assistant.

### Treatment

All patients underwent radical hysterectomy with/without oophorectomy with pelvic lymphadenectomy with/without para-aortic lymphadenectomy. Patients with one or more high-risk factors (lymph node metastasis, parametrial tissue infiltration, positive surgical margin) or two or more moderate risk factors (depth of stromal invasion (DSI) > 1/2, lymphatic vascular stromal infiltration (LVSI), tumour size > 4 cm) were recommended for treatment with sequential chemoradiotherapy (SCRT; 2 courses of chemotherapy were first received, postoperative supplementary radiotherapy was then given 1 week after the end of chemotherapy and then chemotherapy was continued for 2–4 courses after the end of radiotherapy). Postoperative supplementary radiotherapy included external irradiation (1.8–2.0 Gy/time, 5 times/week, with a total dose of 45–50 Gy) and intracavitary radiotherapy (1–2 times/week, with a point dose of 5–10 Gy/week, with a total dose of 35–45 Gy). The dose of external plus intracavitary radiotherapy for the whole course varied with different clinical stages and tumour sizes. The total dose was 75–90 Gy. The chemotherapy regimen was taxol 135 mg/m^2^ intravenous infusion + nedaplatin 80 mg/m^2^ intravenous infusion in a 21-day cycle of 4–6 cycles.

### Follow-up

In this study, all patients were followed up every 3 months for 2 years after surgery, every 6 months for 3 years after surgery and every year for 5 years after surgery. Follow-up was mainly carried out by telephone, outpatient service and electronic medical record system, and the deadline of the follow-up was December 31, 2022. DFS was defined as the time from the date of surgery to recurrence, death, or the last follow-up. Overall survival (OS) was defined as the time from the date of surgery to death or the last follow-up.

### Statistical analysis

SPSS 25.0 was used for data analysis. Age, intraoperative blood loss, LNR and NLR were continuous data, the cutoff values of which were calculated using X-Tile 3.6.1, with death and recurrence as the outcome. Survival curves were plotted using the Kaplan–Meier method, and differences between survival curves were tested with the log-rank test. To determine prognostic factors that affected OS and DFS, we screened all analysis parameters in univariate Cox regression (test level *α* = 0.05), and those with *P* < 0.05 in univariate analysis were subsequently included in the multivariate Cox proportional hazards regression model. In addition, based on the results of multivariate analysis, R 4.2.2 was used to construct a prognostic nomogram to visualize the Cox proportional hazards regression model, and the accuracy of the nomogram was assessed by calculating Harrell’s C-index (0.800 for OS and 0.798 for DFS) and plotting a calibration curve with bootstrapped resampling for 1000 iterations.

## Results

### Patient clinicopathological characteristics

**Fig. 1:**
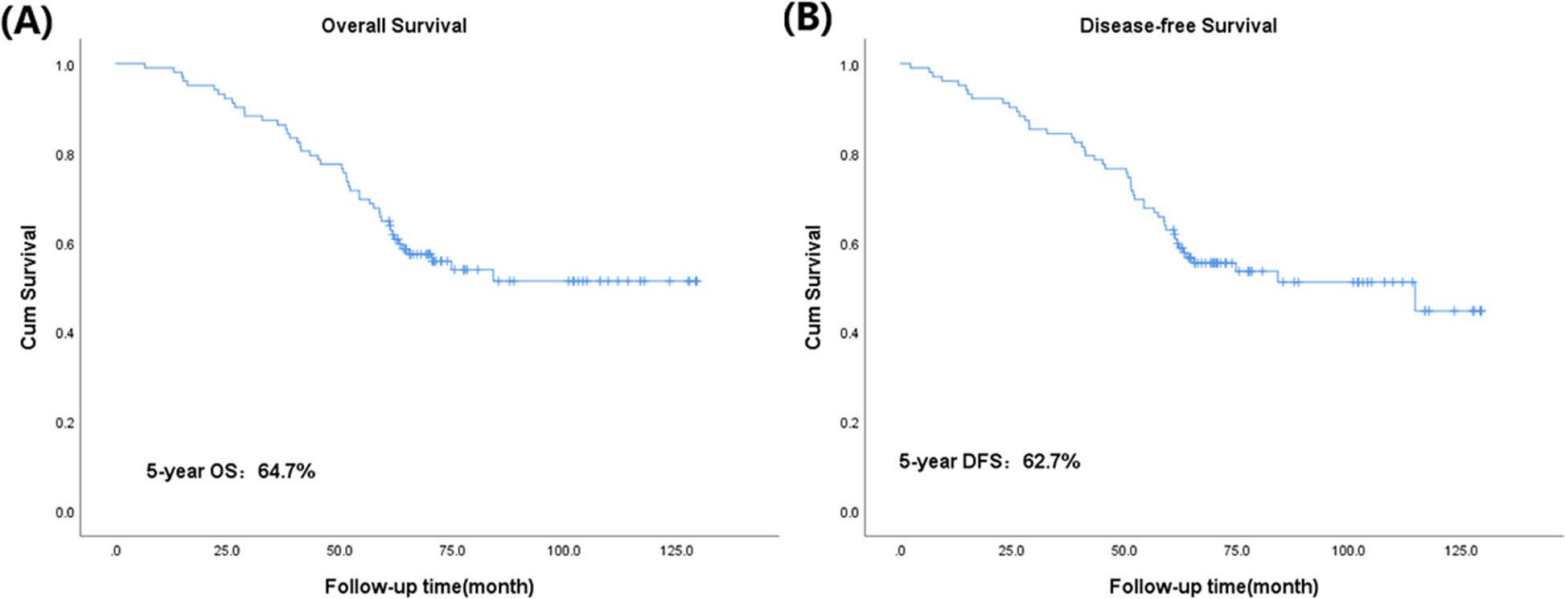
**A** Overall survival in patients with stage IIIC1p cervical cancer. **B** Disease-free survival in patients with stage IIIC1p cervical cancer

**Fig. 2:**
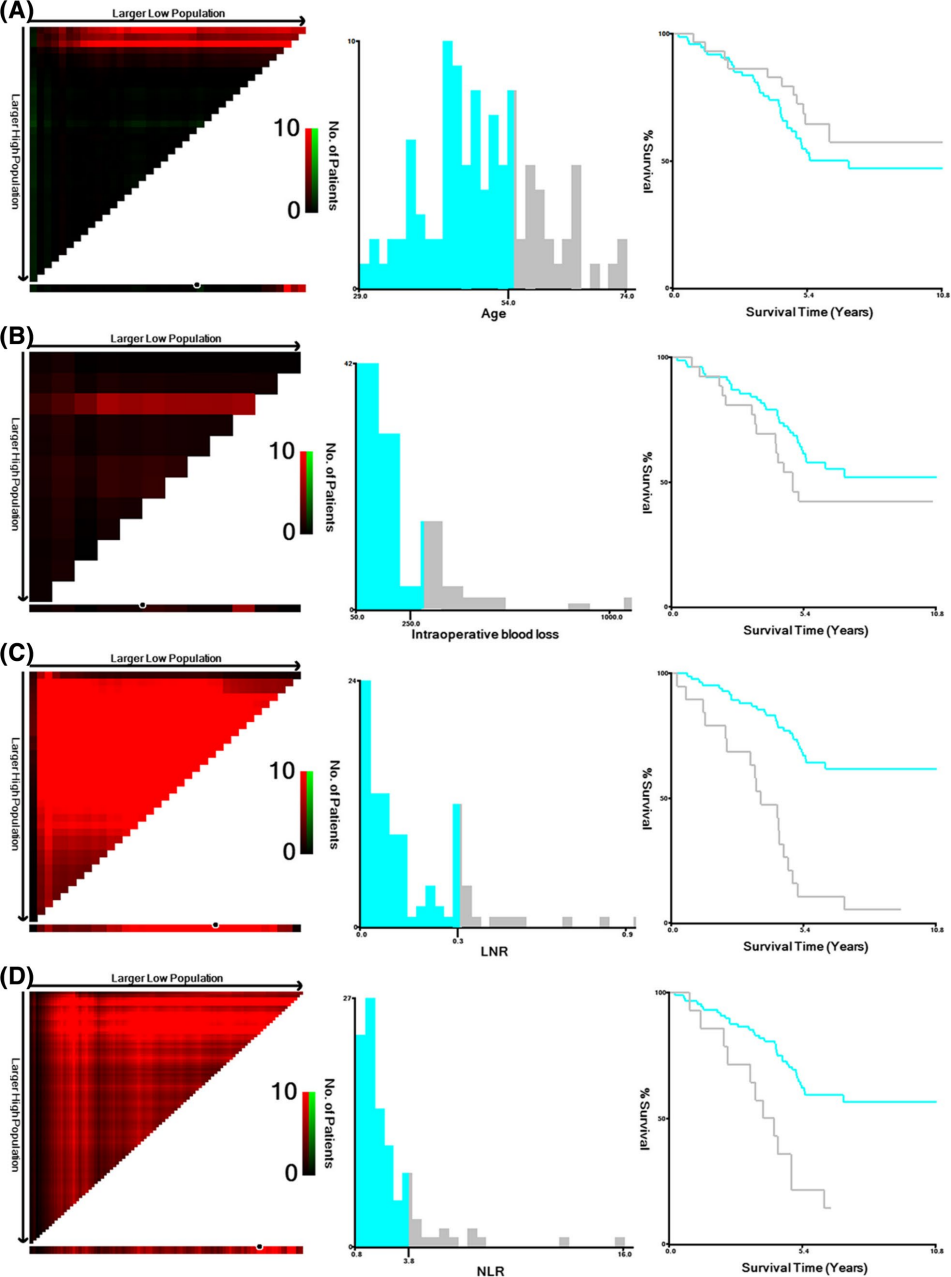
**A** The optimal cutoff value of age calculated by X-Tile software was 54. **B** The optimal cutoff value of intraoperative blood loss calculated by X-Tile software was 250. **C** The optimal cutoff value of LNR calculated by X-Tile software was 0.3. **D** The optimal cutoff value of NLR calculated by X-Tile software was 3.8

A total of 102 patients with pelvic lymph node metastasis confirmed by pathology were included in this study. Ten patients did not receive any treatment after the operation, and 9 patients died by the end of follow-up. Forty patients received single chemotherapy after the operation, with 28 patients dying and 1 patient relapsing by the end of follow-up. Fifty-two patients received complete chemotherapy + radiotherapy + chemotherapy, 9 patients died, and 1 patient relapsed by the end of follow-up. The follow-up time ranged from 6 to 130 months, with a median follow-up time of 63 months. By the end of follow-up, none of the 102 patients in this study were lost to follow-up, with a total of 7 (6.9%) patients relapsing and 46 (45.1%) patients dying. The 5-year OS was 64.7% (Fig. 1A), and the 5-year DFS was 62.7% (Fig. 1B). The optimal cutoff values of age, intraoperative blood loss, LNR and NLR calculated by X-Tile software were 54, 250, 0.3 and 3.8, respectively (Fig. 2). The median number of pelvic lymph node metastases was 3, which was used as the optimal cutoff value. Considering that there may be repetitive variables among the number of metastatic pelvic lymph nodes (mLNs), site of metastatic pelvic lymph nodes (mLN site, unilateral metastasis, or bilateral metastasis) and LNR (the ratio between the number of positive lymph nodes and the total number of lymph nodes removed after surgery), Spearman correlation analysis was performed. The results showed that there was a high correlation among them: mLNs vs. LNR (*p* < 0.001), mLNs vs. mLN site (*p* < 0.001) and mLN site vs. LNR (*p* = 0.001) (Table 1). To avoid the influence of repeated variables on the results, we included only the LNR, a comprehensive factor. The basic information of the patients is shown in Table 2.

**Table 1:** Correlation analysis among mLNs, LNR and mLN site (Spearman)

	*p* value	Correlation Coefficient
mLNs vs. LNR	< 0.001	0.616
mLNs vs. mLN site	< 0.001	0.509
mLN site vs. LNR	0.001	0.337

**Table 2:** Basic information of patients

Variables	Cases, *n* (%)
**Age(years)**	
≤ 54	73 (71.6)
> 54	29 (28.4)
**Surgical method**	
Laparoscope	82 (80.4)
Laparotomy	20 (19.6)
**Postoperative treatment**	
None	10 (9.8)
Chemotherapy	40 (39.2)
Chemo plus radiotherapy	52 (51.0)
**Ovarian status**	
Ovarian transposition	23 (22.5)
Ovariectomy	79 (77.5)
**Intraoperative blood loss(ml)**	
≤ 250	76 (74.5)
> 250	26 (25.5)
**Perioperative complications**	
Negative	76 (74.5)
Positive	26 (25.5)
**Pathological type**	
Squamous cell carcinoma	80 (78.4)
Adenocarcinoma	16 (15.7)
Adeno-squamous carcinoma	6 (5.9)
**Tumor size (cm)**	
≤ 4	61 (59.8)
> 4	41 (40.2)
**LNR**	
≤ 0.3	79 (77.5)
> 0.3	23 (22.5)
**DSI**	
≤ 1/2	10 (9.8)
> 1/2	92 (90.2)
**LVSI**	
Positive	59 (57.8)
Negative	43 (42.3)
**Vagina margin**	
Positive	43 (42.3)
Negative	59 (57.8)
**Parametrium**	
Positive	12 (11.8)
Negative	90(88.2)
**NLR**	
≤ 3.8	88(86.3)
> 3.8	14 (13.7)

*LNR* Lymph node ratio, *DSI* Deep stromal infiltration, *LVSI* Lymph vascular space invasion, *NLR* Neutrophil lymphocyte ratio

### Survival analysis

Univariate Cox regression analysis was performed on all collected factors that could affect the prognosis of patients with stage IIIC1p cervical cancer. The results showed that no adjuvant therapy after surgery, intraoperative oophorectomy, complications with perioperative complications, LNR > 0.3, LVSI, bilateral parametrial involvement and NLR > 3.8 were significantly associated with OS and DFS in patients with stage IIIC1p cervical cancer (*P* < 0.05) (Table 3).

**Table 3:** Univariate analyses of 102 women with 2018 FIGO stage IIIC1p cervical cancer with regard to OS and DFS

Variables	OS		DFS	
	***HR* (95% *CI*)**	***p* value**	***HR* (95% *CI*)**	***p* value**
Age (reference: ≤ 54)	0.738(0.375 ~ 1.454)	0.380	0.688(0.351 ~ 1.350)	0.276
Postoperative treatment (reference: none)	-	-	-	-
Chemotherapy	0.327(0.152 ~ 0.705)	0.004	0.381(0.177 ~ 0.821)	0.014
Chemo plus radiotherapy	0.061(0.024 ~ 0.157)	< 0.001	0.078(0.031 ~ 0.197)	< 0.001
Surgical method (reference: laparoscope)	0.760(0.353 ~ 1.636)	0.483	0.695(0.319 ~ 1.515)	0.361
Ovarian status (reference: ovarian transposition)	2.585(1.090 ~ 6.130)	0.031	2.388(1.060 ~ 5.381)	0.036
Intraoperative blood loss (ml) (reference: ≤ 250)	1.690(0.911 ~ 3.134)	0.096	1.588(0.861 ~ 2.927)	0.139
Perioperative complications (reference: none)	2.277(1.249 ~ 4.150)	0.007	2.102(1.159 ~ 3.813)	0.014
Pathological type (reference: squamous cell carcinoma)	-	-	-	-
Adenocarcinoma	1.197(0.555 ~ 2.581)	0.646	1.098(0.511 ~ 2.359)	0.811
Adeno-squamous carcinoma	1.276(0.392 ~ 4.158)	0.686	1.185(0.364 ~ 3.851)	0.778
Tumor size (cm) (reference: ≤ 4)	1.564(0.875 ~ 2.796)	0.131	1.519(0.861 ~ 2.679)	0.149
LNR (reference: ≤ 0.3)	4.643(2.572 ~ 8.381)	< 0.001	4.490(2.505 ~ 8.050)	< 0.001
DSI (reference: ≤ 1/2)	3.208(0.774 ~ 13.294)	0.108	3.620(0.866 ~ 15.136)	0.078
LVSI (reference: negative)	2.283(1.192 ~ 4.371)	0.013	2.013(1.070 ~ 3.787)	0.030
Vaginal margin (reference: negative)	1.607(0.901 ~ 2.866)	0.108	1.507(0.853 ~ 2.663)	0.158
Parametrium (reference: negative)	2.381(1.108 ~ 5.113)	0.026	2.621(1.264 ~ 5.434)	0.010
NLR (reference: ≤ 3.8)	2.694(1.357 ~ 5.349)	0.005	3.397(1.745 ~ 6.612)	< 0.001

*LNR* Lymph node ratio, *DSI* Deep stromal infiltration, *LVSI* Lymph vascular space invasion, *NLR* Neutrophil–lymphocyte ratio, *CI* Confidence intervals, *HR* Hazard ratio

**Fig. 3:**
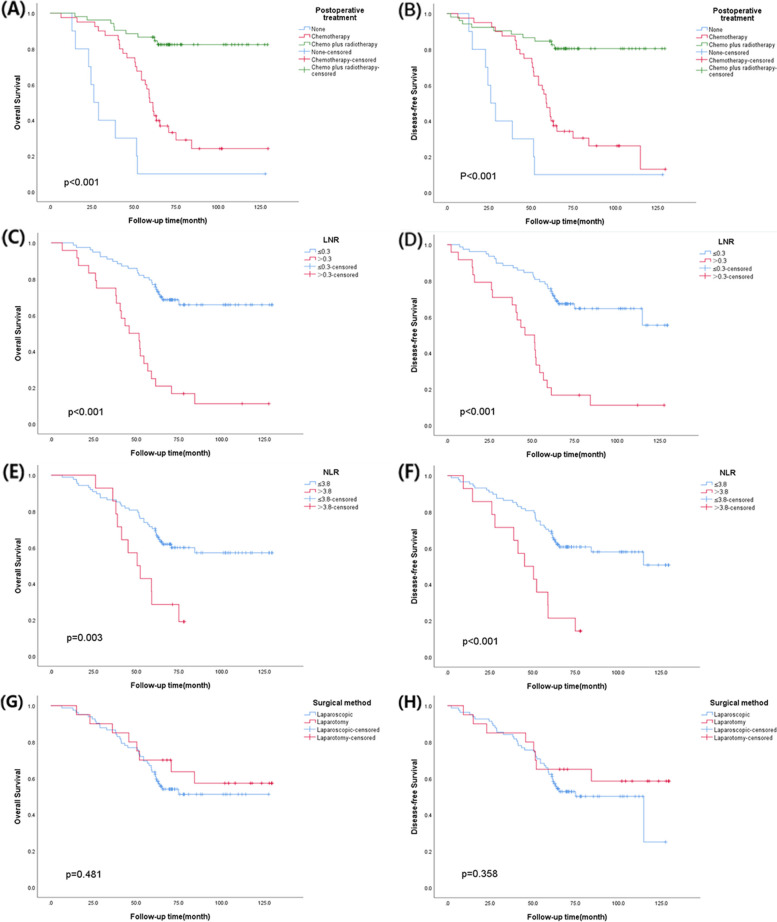
**A** Overall survival curve for postoperative treatment. **B** Disease-free survival curve for postoperative treatment. **C** Overall survival curve for LNR. **D** Disease-free survival curve for LNR. **E** Overall survival curve for NLR. **F** Disease-free survival curve for NLR. **G** Overall survival curve for surgical method. **H** Disease-free survival curve for surgical method. LNR lymph node ratio, NLR neutrophil–lymphocyte ratio

Further multivariate COX regression results showed that no adjuvant therapy, LNR > 0.3 and NLR > 3.8 were independent risk factors for poor OS and DFS in patients with IIIC1p cervical cancer. The detailed results are shown in Table 4. The Kaplan–Meier method was used to draw survival curves and calculate the 5-year OS and 5-year DFS for multivariate analysis results and surgical methods, and the log-rank test was used to test the difference between survival curves (Fig. 3). The 5-year OS and 5-year DFS are shown in Table 5.

**Table 4:** Multivariate analyses of 102 women with 2018 FIGO stage IIIC1p cervical cancer with regard to OS and DFS

Variables	OS		DFS	
	***HR* (95% *CI*)**	***p* value**	***HR* (95% *CI*)**	***p* value**
Postoperative treatment (reference: none)	-	-	-	-
Chemotherapy	0.235 (0.094 ~ 0.587)	0.002	0.357 (0.144 ~ 0.886)	0.026
Chemo plus radiotherapy	0.078 (0.028 ~ 0.220)	< 0.001	0.115 (0.041 ~ 0.321)	< 0.001
Ovarian status (reference: ovarian transposition)	1.730 (0.706 ~ 4.237)	0.231	1.658 (0.708 ~ 3.883)	0.244
Perioperative complications (reference: none)	1.527 (0.753 ~ 3.098)	0.241	1.392 (0.699 ~ 2.773)	0.347
LNR (reference: ≤ 0.3)	4.299 (2.203 ~ 8.389)	< 0.001	3.797 (2.003 ~ 7.197)	< 0.001
LVSI (reference: negative)	1.959 (0.896 ~ 4.282)	0.092	1.481 (0.709 ~ 3.093)	0.296
Parametrium (reference: negative)	1.058 (0.442 ~ 2.532)	0.899	1.369 (0.589 ~ 3.180)	0.466
NLR (reference: ≤ 3.8)	2.129 (1.046 ~ 4.334)	0.037	2.402 (1.208 ~ 4.778)	0.013

*LNR* Lymph node ratio, *LVSI* Lymph vascular space invasion, *NLR* Neutrophil–lymphocyte ratio, *CI* Confidence intervals, *HR* Hazard ratio

**Table 5:** Five-year OS and 5-year DFS for patients in different groups

Variables	5-year OS	5-year DFS
**Postoperative treatment**		
None	10.0%	10.0%
Chemotherapy	50.0%	47.5%
Chemo plus radiotherapy	86.5%	84.6%
**LNR**		
≤ 0.3	76.9%	75.6%
> 0.3	25.0%	20.8%
**NLR**		
≤ 3.8	70.5%	69.3%
> 3.8	28.6%	21.4%
**Surgical method**		
Laparoscope	63.4%	62.2%
Laparotomy	70.0%	65.0%

*LNR* Lymph node ratio, *NLR* Neutrophil–lymphocyte ratio, *OS* Overall survival, *DFS* Disease-free survival

### Nomogram

**Fig. 4:**
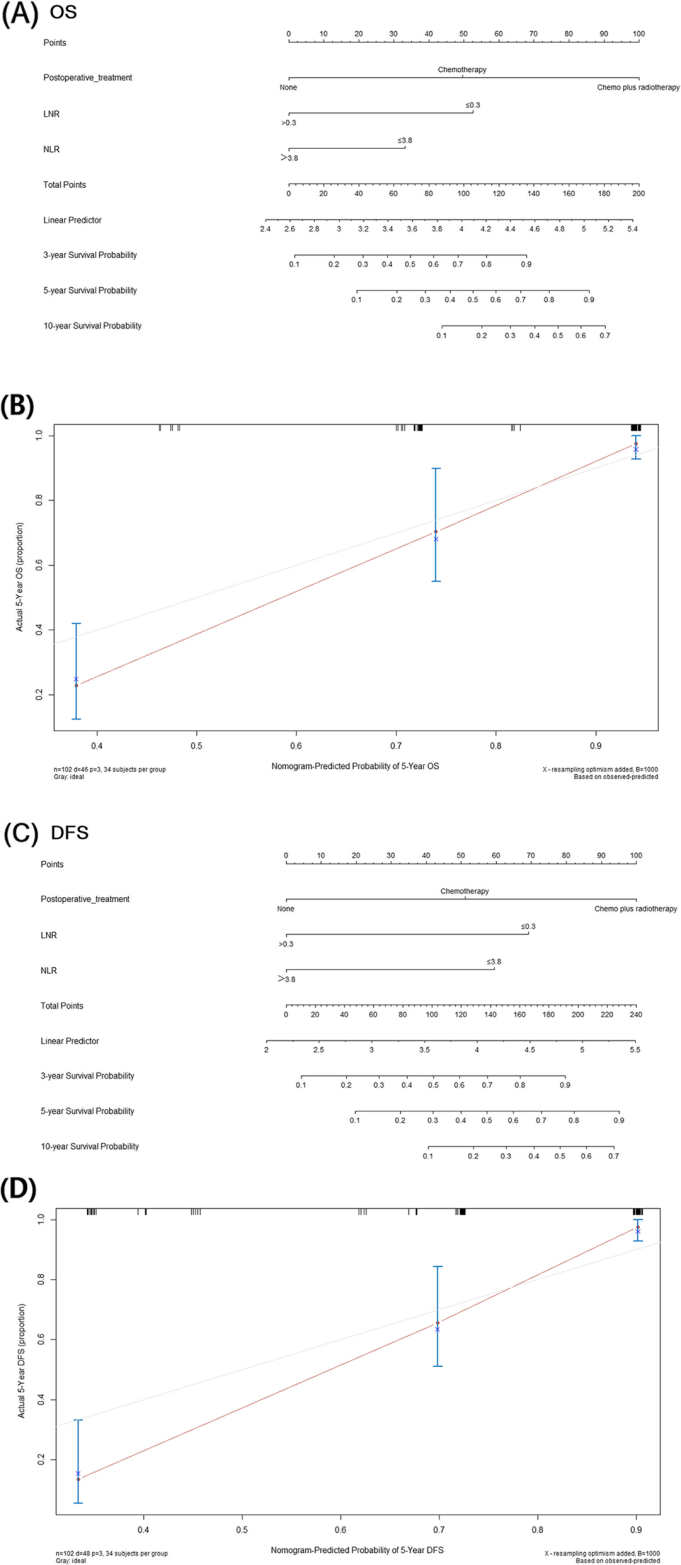
**A** Nomogram for predicting OS, which had a C-index of 0.800. **B** Calibration curve to predict 5-year OS. **C** Nomogram for predicting DFS, which had a C-index of 0.798. **D** Calibration curve to predict 5-year DFS. OS overall survival, DFS disease-free survival, NLR neutrophil–lymphocyte ratio

Subsequently, we created nomograms based on the three variables of postoperative adjuvant therapy (no adjuvant therapy versus single chemotherapy versus chemotherapy plus radiotherapy), LNR (LNR ≤ 0.3 versus LNR > 0.3) and NLR (NLR ≤ 3.8 versus NLR > 3.8) to visualize the Cox proportional hazard regression model to better predict OS and DFS in patients with stage IIIC1p cervical cancer (Fig. 4A, C); the C indexes were 0.800 and 0.798, respectively. The calibration curves of 5-year OS and 5-year DFS are shown in Fig. 4B and D, respectively, demonstrating good consistency.

## Discussion

Cervical cancer is the fourth most common cancer in women worldwide, accounting for nearly 8% of all cancer deaths in women each year [10]. Studies have shown that lymph node metastasis can occur in the early stage of cervical cancer, with an incidence of 10% to 20% [11]. Compared with patients without lymph node metastasis as confirmed by pathology after surgery (5-year survival rate can be as high as 90%), the 5-year survival rate of patients with positive pelvic lymph nodes is reduced to 50 ~ 60% [12]. All 102 patients included in this study had pelvic lymph node metastasis after radical hysterectomy, with a 5-year OS of 64.7% and a 5-year DFS of 62.7%, which is consistent with previous studies. The new 2018 FIGO cervical cancer staging system classifies patients with pelvic lymph node metastasis as stage IIIC1 cervical cancer, which undoubtedly reflects the importance of pelvic lymph node metastasis to the prognosis of cervical cancer patients, and the treatment method has also been changed accordingly. However, the rationality of this stage is still controversial.

According to the 2009 FIGO staging system, radical hysterectomy and postoperative adjuvant therapy are recommended for patients with pelvic lymph node metastasis in stage IA ~ IIA, and studies have shown that such patients have a good prognosis. In the new 2018 FIGO staging system, direct radical concurrent chemoradiotherapy is recommended for stage IIIC1, but some experts believe that some patients in stage IIIC1 can also receive radical surgery plus chemoradiotherapy [13]. A retrospective study by Suprasert et al. [14] revealed that patients who gave up radical surgery and switched to radical chemoradiotherapy due to positive lymph nodes during surgery had a worse prognosis than those who completed radical surgery plus postoperative supplementary chemoradiotherapy (2-year DFS was 93.5%), with a 2-year DFS of only 58.5%. A multicentre retrospective study by Derks et al. [15] also reached the same conclusion. Moreover, the rationality of imaging staging is controversial. Ferrandina et al. [16] found that the sensitivity of MRI and PET-CT in detecting preoperative lymph node metastasis was only 35.7% versus 28.6%, and the specificity and accuracy were 95.9% versus 97.8% and 88.0% versus 88.7%, respectively.

Therefore, considering the favourable effect of surgical treatment on prognosis and the uncertainty of imaging, we sought to analyse the factors affecting the prognosis of patients with stage IIIC1p cervical cancer treated by surgery. Therefore, 102 patients (preoperative imaging showed no lymph node metastasis) with pelvic lymph node metastasis confirmed by pathology after radical hysterectomy were included as the homogeneous group, and additional radiotherapy and chemotherapy were recommended after surgery. Univariate analysis showed that no postoperative adjuvant therapy was associated with poor OS and DFS in patients with stage IIIC1p cervical cancer. The 5-year OS and DFS of patients with no postoperative adjuvant therapy, single chemotherapy and completed radiotherapy + chemotherapy + radiotherapy were 10.0% versus 10.0%, 50.0% versus 47.5% and 86.5% versus 84.6%, respectively. Multivariate analysis showed that no postoperative adjuvant therapy was an independent risk factor for poor OS and DFS in patients with stage IIIC1p cervical cancer. Huang et al. [17] found that the distant recurrence-free survival rate in the SCRT group after radical hysterectomy was significantly higher than that in the concurrent chemoradiotherapy (CCRT) group and single treatment group. In this study, 5-year OS and 5-year DFS were nearly doubled in patients who completed full-course SCRT compared with those who received chemotherapy only, reflecting the role of SCRT in reducing recurrence and distant metastasis. However, as this study was conducted in a single institution and the number of patients was limited, the sample size should be further expanded for follow-up studies.

Wright et al. [18] analysed the prognosis of cervical cancer patients in the national cancer database and found that the survival rate of patients with pelvic lymph node metastasis (stage IIIC1) was higher than that of patients with stage IIIA and IIIB, even closer to that of patients with stage II. This also reflects the heterogeneity of prognosis in patients with stage IIIC1 cervical cancer. Therefore, it may be limited to include only pelvic lymph node metastasis in stage IIIC1. Some parameters related to lymph node status have been reported in the literature, such as the number of metastatic lymph nodes, the total number of resected lymph nodes and the location of positive lymph nodes [19, 20]. However, different studies have supported different conclusions. Theoretically, the number of metastatic lymph nodes depends directly on the number of lymph nodes resected [21]. Zhou et al. [22] suggested that increasing the number of lymph node resections could reduce the risk of occult metastasis. However, the minimum number of lymph nodes to be resected to ensure the quality of resected lymph nodes is still a controversial issue [23].

LNR, as included in the study, is a relatively new prognostic factor. It is a comprehensive factor that refers to the ratio between the number of positive lymph nodes and the total number of lymph nodes removed after surgery [24]. It is related to both metastatic lymph node burden and whether lymph node resection is complete. It enables more accurate assessment of the status of lymph nodes and better stratification of patient prognosis. LNR has been shown to be an independent predictor of pancreatic cancer [25], gastric cancer [26] and breast cancer [27]. At present, researchers in several retrospective studies have reported the relationship between LNR and survival outcome of cervical cancer patients, but the conclusions are not the same. The value of the LNR in predicting the survival of cervical cancer patients is still controversial [21, 28, 29]. Considering that LNR may be higher when the total number of resected pelvic lymph nodes is limited, it may not accurately reflect tumour burden. Therefore, the minimum total number of pelvic lymph nodes removed in our study was 12. According to the results of previous studies, the cutoff value of LNR in patients with cervical cancer varies in the range of 0.05–0.4 [21, 30]. Our study was focused on stage IIIC1p cervical cancer patients, with death and recurrence as the outcome, and the cutoff value of LNR calculated by X-Tile software was 0.3, which also fell within this range. The univariate log-rank test and univariate COX analysis showed that LNR > 0.3 was associated with poor OS and DFS, with 5-year OS and 5-year DFS rates of 25.0% and 20.8%, respectively. The 5-year OS and 5-year DFS of patients with LNR ≤ 0.3 were 76.9% and 75.6%, respectively. Multivariate analysis showed that LNR > 0.3 was an independent risk factor for OS (HR: 4.229, 95% CI: 2.203 ~ 8.389, *p* < 0.001) and DFS (HR: 3.797, 95% CI: 2.003 ~ 7.197, *p* < 0.001) in patients with stage IIIC1p cervical cancer. Some scholars believe that patients with a higher LNR often have more cancer cells in their lymph nodes, so the probability of increasing fragmented and scattered cancer tissues or cancer cells in the pelvic and abdominal cavities during surgical resection is greater [28]; this may be one of the reasons for the poor prognosis of patients with stage IIIC1p cervical cancer. This conclusion further supports our research results. Therefore, the LNR has good prognostic value for patients with stage IIIC1p cervical cancer.

Recently, an increasing number of studies have shown that inflammatory markers play a key role in the prognosis of various malignant tumours [31]. The NLR is one of the markers of the systemic inflammatory response [32] and has been reported to be associated with poor prognosis in various cancers, such as hepatocellular carcinoma [33], gastric cancer [34] and breast cancer [35]. Similarly, the NLR has been shown to have prognostic value in patients with cervical cancer [36, 37]. This retrospective analysis shows that an NLR > 3.8 is an independent risk factor for poor OS and DFS in patients with stage IIIC1p cervical cancer, summarizing a result that is consistent with previous studies. The underlying mechanism primarily involves the systemic inflammatory response from cancer cells promoting neutrophil infiltration, which subsequently promotes cancer progression through the secretion of interleukin-2 (IL-2), IL-6, IL-10, tumour necrosis factor-α and vascular endothelial growth factor. As cancer progresses, the body’s immune system is compromised, and lymphocytes are reduced [32]. Therefore, preoperative measurement of the NLR has guiding value for the prognosis of patients with stage IIIC1p cervical cancer.

Notably, the publication of the LACC trial results has led to a major shift in the surgical treatment of cervical cancer. Bogani et al. [38] analysed patients who underwent radical hysterectomy before and after the LACC trial and found that the number of patients who underwent minimally invasive radical hysterectomy decreased from 64.9 to 30.4% (*p* < 0.001), while there was no significant difference in 90-day surgery-related morbidity among patients who underwent radical hysterectomy before and after the LACC trial (18.9% versus 16.6%, *p* = 0.795). This also confirms the results of the LACC trial, which showed no significant difference in surgery-related morbidity between the two approaches [39]. However, the adverse effects of minimally invasive surgery on survival cannot be ignored. We now live in the era of post-LACC, so the influence of surgical method on survival outcomes should be considered in the study. In a retrospective study, Kim et al. [28] found that the poor prognosis in the high LNR group was aggravated by laparoscopic surgery, which may be related to the fact that laparoscopic surgery made it easier for positive pelvic or para-aortic lymph nodes to be decomposed into microscopically small units and thus more easily exfoliated. In a multicentre retrospective study, Bogani et al. [40] found that patients undergoing laparoscopic radical hysterectomy had a higher risk of developing intrapelvic recurrence and peritoneal carcinomatosis than those undergoing open surgery. This higher risk may be related to the use of a uterine manipulator, cancer cells contaminating the pelvis at the time of colpotomy, CO2 promoting the spread of cancer cells in mechanical and biochemical ways and CO2 pressure promoting the infiltration of cancer cells into the superficial mesothelial layer of the peritoneum [38, 41]. In this study, we applied univariate log-rank test and univariate COX regression analysis and showed that open surgery and laparoscopic surgery had no significant effect on OS and DFS in patients with IIIC1p cervical cancer. The 5-year OS and 5-year DFS of patients undergoing laparoscopic surgery were 63.4% and 62.2%, respectively, and those of patients undergoing open surgery were 70.0% and 65.0%, respectively. The small number of patients undergoing abdominal radical hysterectomy may bias the results. Thus, the sample size should be expanded for further studies to explore the impact of surgical methods on the prognosis of patients with stage IIIC1p cervical cancer and the correlation between surgical methods and LNR.

The current study has several limitations. First, the study was retrospective, and not all patients received postoperative adjuvant therapy. Second, this study was conducted in a single institution, the sample size was small, and the conclusions may not be universal. Third, LNR is directly related to the number of metastatic lymph nodes and the total number of lymph nodes resected, both of which depend on the extent to which the pathologist examines the surgical specimen. Fourth, the cases included in this study were from 2012 to 2017, and the patients who underwent laparoscopic surgery accounted for a large proportion. To better study the effect of laparoscopic surgery and open surgery on the prognosis of patients with cervical cancer, the sample size needs to be further expanded. Fifth, the grade of cervical cancer was not included in this study, and future studies should also assess the impact of cervical cancer grade on the prognosis of patients. It should be noted that no patient was lost to follow-up in this study, and the follow-up time was 6–130 months, with a median follow-up time of 63 months, which means that the survival information of patients was relatively complete.

## Conclusion

Our study shows that there is heterogeneity in the prognosis of patients with stage IIIC1p cervical cancer. The factors of no postoperative adjuvant therapy after radical hysterectomy, LNR > 0.3 and NLR > 3.8 have significant effects on the prognosis of patients with stage IIIC1p cervical cancer. In clinical work, comprehensive assessment of the above factors can be of value to clinicians as they more actively treat and manage patients.

## Data Availability

The datasets used and/or analysed during the current study are available from the corresponding author on reasonable request.

## Abbreviations

CC: Cervical cancer
CCRT: Concurrent chemoradiotherapy
CI: Confidence intervals
CT: Computerized tomography
DFS: Disease-free survival
DSI: Depth of stromal invasion
ESGO/ESTRO/ESP: The European Society of Gynaecological Oncology/European Society for Radiotherapy and Oncology/European Society of Pathology
FIGO: Federation International of Gynecology and Obstetrics
HR: Hazard ratio
LACC: The Laparoscopic Approach to Cervical Cancer
LNR: Lymph node ratio
LVSI: Lymphatic vascular stromal infiltration
mLNs: Metastatic pelvic lymph nodes
MRI: Magnetic resonance imaging
NCCN: The National Comprehensive Cancer Network
NLR: Neutrophil-lymphocyte ratio
OS: Overall survival
PET-CT: Positron emission tomography-Computerized tomography
SCTR: Sequential chemoradiotherapy
